# Rubus idaeus extract improves symptoms in knee osteoarthritis patients: results from a phase II double-blind randomized controlled trial

**DOI:** 10.1186/s12891-022-05612-2

**Published:** 2022-07-07

**Authors:** Yves Henrotin, Romain Le Cozannet, Pascale Fança-Berthon, Romain Truillet, Martine Cohen-Solhal, Gillian DunnGalvin, Jean-Marie Grouin, Andrea Doolan

**Affiliations:** 1 musculoSKeletal Innovative Research Lab (mSKIL), The Center for Interdisciplinary Research On Medicines (CIRM), Department of Motricity Center, Institute of Pathology, University of Liège, CHU Sart-Tilman, level 5, 4000 Liège, Belgium; 2 Department of Physical Therapy and Rehabilitation, Princess Paola Hospital, Vivalia, Marche-en-Famenne, Belgium; 3 Artialis SA, GIGA Tower, CHU Sart-Tilman, Avenue de l’hôpital, 4000 Liège, Belgium; 4 Naturex SA, Avignon, France; 5 Atlanstat SA, Les Espaces Océane, 3, rue Jules Verne, 44400 Rezé, France; 6 BIOSCAR Inserm U1132 and Department of Rheumatology, Université de Paris, Hôpital 10 Lariboisière, 75010 Paris, France; 7 Atlantia Clinical Trials, Heron House Offices, Blackpool, T23 R50R Cork Ireland; 8 Department of Statistics, Rouen University, Rouen, France

**Keywords:** Osteoarthritis, Knee, Rubus idaeus, Pain, Clinical trial

## Abstract

**Background:**

Osteoarthritis (OA) is the most frequent cause of disability in elderly people. In daily practice, the main objective of the physician is to reduce patient symptoms using treatments without adverse effects. However, the most prescribed treatment to manage OA symptoms remains nonsteroidal anti-inflammatory drugs which are associated with severe adverse effects. Therefore, we need a safe alternative to managing OA. One candidate is Rubus idaeus leaf extracts known to inhibit inflammatory responses.

**Objective:**

This study aimed to evaluate the effects of a 12-weeks intervention with an ethanolic extract from Rubus idaeus leaf on symptoms of knee osteoarthritis.

**Method:**

The study was a randomized, double-blind, placebo-controlled, monocentric trial of 198 participants with femorotibial osteoarthritis. Participants were randomized equally to receive one daily during 3 months either 1 capsule of Rubus idaeus leaf extract 400 mg, 1 capsule of Rubus idaeus leaf extract 200 mg, or 1 capsule of placebo. The participants were assessed at baseline and after one and three months of treatment. The primary endpoint was an absolute change of the Western Ontario McMaster osteoarthritis index (WOMAC) pain subscale. The secondary endpoints were WOMAC global score, stiffness and function sub-scales, knee pain VAS score at walking, the Short Form (SF)-36, the Short Physical Performance Battery (SPPB), the 20-m walk test, and the International Physical Activity Questionnaire (IPAQ) and Outcome Measures in Rheumatology Clinical Trials and Osteoarthritis Research Society International (OMERACT-OARSI) responders rate. Statistical analyses were conducted on the intent-to-treat (ITT) population.

**Results:**

In the Intention-to-treat population, WOMAC pain was not significantly modified by Rubus idaeus leaf extract compared to placebo. In contrast, Rubus idaeus leaf extract 400 mg after 12 weeks of treatment significantly reduced pain measured by the VAS. The mean pain decrease induced by Rubus ideaus leaf extract was over -7 mm which is clinically relevant and reached a clinically statistical difference compared to placebo with the highest dose. Rubus Ideaus was not significantly more efficient than the placebo on WOMAC global score, stiffness, and physical function subscores, IPAQ, SF-36, walking distance in treadmill test, SPPB, and evaluation of associated treatments needed to manage OA.

**Conclusion:**

Rubus idaeus leaf extract was well tolerated and effective to relieve pain in a patient with knee osteoarthritis.

**Trial registration:**

NCT03703024 (11/10/2018).

**Supplementary Information:**

The online version contains supplementary material available at 10.1186/s12891-022-05612-2.

## Introduction

Osteoarthritis (OA) is the major cause of disability in older adults. In the USA, Federal Drug Administration (FDA) has recognized that OA can be a serious disease for which no pharmacological treatment can modify the underlying pathophysiology of the disease and change its natural course [1]. Currently, patient management aims to reduce symptoms and improve quality of life using therapeutic modalities with low side effects. Recently, the Osteoarthritis Research Society International (OARSI) recommended patient education, physical activities including exercise programs, and weight control with diet intervention as the core treatment for all patients whatever their health status and OA severity [2]. Pharmacological modalities can be associated with core treatment if the core treatment alone is not satisfying or to facilitate patients' adhesion to exercise programs. Among these pharmacological modalities, the use of Nonsteroidal Anti-Inflammatory Drugs (NSAIDS) was recommended in well-defined conditions while opioids and paracetamol were no more recommended. Therefore, there is a need for safe treatments with an efficacy supported by well-conducted clinical trials. One safe approach could be nutraceuticals for which the most used to manage joint discomfort are glucosamine, chondroitin, collagen, Boswellia, and turmeric extracts [3]. Another potential candidate is Rubus idaeus (Raspberry) leaf extract (RIE) rich in flavonoids and phenols that are known to inhibit inflammatory responses [4] by preventing the activation of MAPK or NFkB signaling pathways [5]. Moreover, polyphenolic-enriched red raspberry Rubus fruit extract reduces collagen breakdown in bovine chondrocytes as well as the severity of arthritis in an antigen-induced arthritis rat model [6]. In cartilage explants, RIE prevented the loss of proteoglycan and MMP-3 and MMP-13 protein expressions. RIE reduced the expression of interleukin (IL)-1 and -6 in macrophages, without change in Tumor Necrosis Factor (TNF) and cyclooxygenase (Cox)-2 expression. The secretome of macrophages pre-treated with RIE and transferred in chondrocytes decreased the gene expression and protein synthesis of MMP-3, -13, and Cox-2. Globally, these in vitro studies suggested that RIE could limit synovitis and prevent cartilage degradation without inducing toxicity [4]. Patented data (US20200222486A1) demonstrated that RIE increased the secretion of 5-HETE and 12-HETE, two intermediaries of the lipoxygenase pathway involved in the resolution of inflammation-induced in mice by injection of methylated bovine serum albumin (mBSA). In this model, Rubus Idaeus extract decreased the level of circulating IL-6 plasma but increased the level of 12-HETE, as well as reduced joint swelling in paws [7]. No toxic effects of the investigational product have been reported.

In this paper, we report the effect of RIE, on symptoms impairing the quality of life in people suffering from OA. Thus, we conducted a randomized, double-blinded, placebo-controlled trial to assess whether supplementation with RIE improved OA knee pain and function. This study was the first trial applied to the human body. Further, this phase II study has been conducted in full accordance with the International Council for Harmonisation of Technical Requirements for Pharmaceuticals for Human use (ICHE6) that examined the impact of ingesting a food supplement composed of RIE on alleviating pain, function, and physical performance in elderly participants who reported having mild to moderate knee pain.

## Population and design

### Study design

This study was a phase II randomized, double-blind, placebo-controlled with three parallel groups and a monocentric trial including 195 patients with a primary knee OA. Participants were recruited from June 1, 2017, to December 31, 2018.

The main inclusion criteria were an age of 30 to 75 years, a Body Mass Index (BMI) between 18.5 and 35 kg/m^2^, a documented diagnosis of primary OA of the target knee made at least 12 months before screening, radiographic evidence of OA in the tibiofemoral compartment of the target knee with at least one osteophyte and a measurable joint space narrowing, as diagnosed by standard X-rays taken no longer than 18 months, and a mild to moderate pain not adequately or completely controlled with NSAIDs. The most painful knee was considered the target knee. The main exclusion criteria were pregnancy or lactation, secondary knee OA, a Kellgren-Lawrence grade IV in the patellofemoral compartment of the target knee, a clinically objective effusion of the target knee or other joint, asymptomatic OA of the contralateral knee that was not responsive to paracetamol and required other therapy, change of dietary habit within the preceding month, allergy or contraindication to the tested product, a concurrent medical or psychiatric condition that, in the opinion of the investigator, could have compromised patient's ability to comply with the study requirements, use of viscosupplementation in any joint including the target knee or other joint within 9 months before screening. Calcium or other dietary supplements in the last months were also exclusion criteria. Participants enrolled could have taken paracetamol and/or oral NSAIDs to manage knee pain. Participants were then asked to use these rescue medications only when needed during the trial. Twenty-four hours before a visit, participants were asked to stop rescue medication for the evaluation of clinical parameters by the investigator. The trial has been conducted following the Good Clinical Practices (GCP) guidelines and according to the "Declaration of Helsinki" published by the World Medical Association. The study protocol was approved by the Central Ethics Committee of The University College Cork, Irland (namely Clinical Research Ethics Committee), agreement number: ECM 4 (I) 07/02/17.

This RCT was also registered on Clinical trial.gov on NCT03703024 https://clinicaltrials.gov/ct2/show/NCT03703024 (first registration date 11/10/2018).

### Treatment assignment

The participants were randomly assigned to one of the study groups. They received one daily each morning for 12 weeks either 1 capsule containing 400 mg of RIE or 1 capsule containing 200 mg of RIE or 1 capsule of placebo. RIE was a natural hydro-alcoholic extract produced from the leaves of Rubus Idaeus accordingly to patent US20200222486A1. The RIE was standardized in polyphenols such as sanguiine H6 (C82H54O52; molar mass: 1871.27 g / mol) which is one of the main active ingredients. The placebo capsules contained 100% maltodextrin.

### Outcome measures

The primary outcome was changed, if any, in pain scores in the target knee joint from baseline to the end of treatment using WOMAC Western Ontario and McMaster Universities Osteoarthritis Index Likert Scale Version 3.1 (WOMAC LK 3.1) pain subscale with a possible score range between 0 to 20.The secondary outcomes were pain change using the VAS (Visual Analogue Scale), change in the WOMAC global (sum of each WOMAC subscale) or stiffness ranging from 0 to 8 and physical function ranging from 0 to 68 subscales, and the participant global assessment of the quality of life using a short form (SF) survey of 36 questions, the International Physical Activity Questionnaire (IPAQ) expressed as MET-min per week, the 20-m walking speed, the Short Physical Performance Battery (SPPB) including gait speed measured over 3 m, chair stand time, and standing balance evaluation, the evaluation of associated treatments needed to manage OA and the Outcome Measures in Rheumatology Clinical Trials and Osteoarthritis Research Society International (OMERACT-OARSI) responders rate. The OMERACT–OARSI criteria for response are (1) improvement in pain or physical function ≥ 50% and an absolute change ≥ 20 mm; or (2) improvement of ≥ 20% with an absolute change ≥ 10 mm in pain and physical function. Compliance with the study treatments was established by counting unused study products. All variables were recorded at baseline, after 6 weeks and 12 weeks of treatment.

### Determination of the sample size

A prior power calculation was used to determine the sample size in this trial. To determine the appropriate sample size a literature review was completed. The analysis of different nutraceutical medication studies with a similar primary endpoint in the OA population showed a significant response to treatment using a group size of an average of *n* = 45 patients [8, 9]. Taking this information into consideration a sample size of 60 patients/group (*n* = 180) was chosen to be more than adequate to meet sample size requirements defined for a decrease of 14% of the WOMAC scores taking into account a drop-out rate of 7–9% for a treatment period of 3 months, an α of 0.05 and a β of 0.20 (power of 80%). With an expected 8% drop-out rate, it was decided that 65 participants were to be randomized into each group.

### Statistical analysis

Statistical analyses were conducted by using SAS version 9.4 (SAS Institute, Cary, NC) on the intent-to-treat (ITT) population, which included all participants who were randomized into the study, and consumed at least one dose of the study product. Between-group assessments at baseline were evaluated by one-way-analysis-of-variance (ANOVA) for continuous data and Chi-Square for independence for categorical data. In a posthoc analysis, participants with BMI < 25 or ≥ 25 kg/m^2^ have been compared. The cut-off of 25 was selected because over this value the patient fell within the overweight or obese range. Change from baseline was used for comparisons. Change scores were evaluated by mixed-model repeated-measures analysis containing the treatment group, the visit, the baseline score of dependent outcomes, and the treatment x visit interaction. The adequacy of the model was verified by residuals analysis. Normality distribution of the residuals was verified by Skewness and Kurtosis (less than 2 in absolute value). Data presented were the mean and standard error model (SEM). All tests of significance were completed at α = 0.05, two-tailed. Dunnett corrections were performed to adjust the p-value for multiple comparisons (active treatment group versus a placebo group).

## Results

### Population

**Fig. 1:**
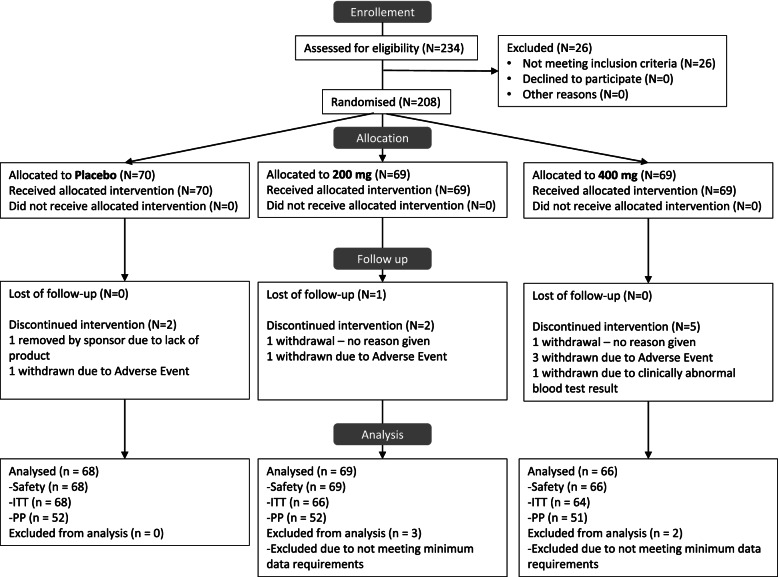
Flow diagram of RUBUS study

A total of 208 participants (74 men and 124 female) were randomly assigned to treatment on a 1:1:1 basis, where *n* = 70 participants were allocated to the Placebo arm, *n* = 69 participants were allocated to the 200 mg RIE arm, and *n* = 69 participants were allocated to the 400 mg RIE arm. Nine participants withdrew prematurely from the study, five of these were withdrawn due to an adverse event/Severe Adverse Event (SAE), one participant was withdrawn due to receiving a clinically abnormal blood result, and one participant was withdrawn due to a lack of study product, and two participants did not give reasons for their withdrawal. All other 198 participants completed the study as planned (Fig. 1).

Two hundred three participants were included in the Safety population as they took at least one dose of the product, 198 participants were considered eligible for the ITT analysis. Among the Safety population, 68 participants received a placebo, 69 RIE 200 mg, and 66 RIE 400 mg. The number of withdrawals was 2 in the placebo group, 2 in the RIE 200 mg group, and 5 in the 400 mg group (Fig. 1; Additional file 1). At baseline, participants in each group were well-matched (Table 1). Females and males were equally distributed among the three groups. 68.7% in RIE 400 mg, 74.3% in RIE 200 mg, and 70.6% of placebo participants were overweight or obese (BMI ≥ 25 kg/m2). All participants had a diagnosis of OA. 53% had a Kellgren-Lawrence score of 1 and 38% had a Kellgren-Lawrence score of 2. The remaining participants (9%) had a Kellgren-Lawrence score of 3. No significant differences were observed between the three treatment groups according to demographic characteristics and BMI. Fifty-one participants had a BMI < 25 kg/m2 and 147 had a BMI ≥ 25. At baseline, global WOMAC and subscores, VAS pain, SF-36, SPPB, and IPAQ scores were not significantly different between BMI groups (Additional file 2).

**Table 1:** Demographic and baseline characteristics (ITT population *N* = 198))

		**Placebo (*N* = 68)**	**200 mg** **(*N* = 66)**		**400 mg** **(*N* = .64)**	**All** **(*N* = 198)**	***P*-values**
**Sex**	n/n miss	68/0	66/0		64/0	198/0	0.52
	Male, n(%)	25 (36.8%)	28(42.4%)		21(32.8%)	74 (37.4%)	
	Female, n(%)	43(63.2%%)	38(57.6.%)		43 (67.2%)	124 (62.6%)	
**Ethnicity**	n/n miss	68/0	66/0		64/0	198/0	0.37
	Asian, n(%)	0(0.0%)	1(1.5%%)		0 (0.0%)	1 (0.5%%)	
	African, n(%)	0 (0.0%)	0(0.0%)		0 (0.0%)	0 (0.0%)	
	Caucasian, n(%)	68(100.0%)	65 (98.5%)		64 (100.0%)	197(99.5%)	
**Age (years)**	n/n miss	68/0	66/0		64/0	198/0	0.21
	Mean (SEM)	52.93 (1.39)	55.71 (1.21)		55.69 (1.25)	54.75 (0.75)	
	Median	52.50	54.50		56.60	55.00	
	Min, Max	30.0, 74.0	31.0, 75.0		35.0, 74.0	30.0, 75.0	
**BMI (kg/m**^**2**^**)**	n/n miss	68/0	66/0		64/0	198/0	
	Mean (SEM)	27.16 (0.40)	26.70 (0.36)		26.88 (0.41)	26.92 (0.22)	
	Median	27.49	26.60		26.49	26.69	0.70
	Min, Max	20.10, 32.20	19.96, 31.83		19.06, 33.88	19.06, 33.88	
	Normal [18.5—25[ Overweigth [25–30[ Obese ≥ 30	20 (29.4%) 32 (47.1%) 16 (23.5%)	17 (25.8%) 38 (57.6%) 11 (16.7%)		20 (31.3%) 31 (48.4%) 13(20.3%)	57 (28.8%) 101 (51.0%) 40 (20.2%)	
**WOMAC global**	n/n miss	68/0	66/0		64/0	198/0	0.81
	Mean (SEM)	24.43 (1.68)	25.84(1.47)		24.80(1.58)	25.02 (0.91)	
	Median	20.83	26.04		23.44	22.92	
	Min, Max	3.1, 63.5	4.2, 55.2		6.3,56.3	3.1, 63.5	
**WOMAC pain**	n/n miss	68/0	66/0		64/0	198/0	0.64
	Mean (SEM)	4.66 (0.34)	5.14 (0.38)		4.87 (0.36)	4.89 (0.21)	
	Median	4.00	5.00		4.00	4.00	
	Min, Max	1.0, 14.0	0.0, 16.0		1.0, 12.0	0.0, 16.0	
**WOMAC stiffness**	n/n miss	68/0	66/0		64/0	198/0	0.94
	Mean (SEM)	2.71 (0.19)	2.77 (0.17)		2.78 (0.16)	2.75 (0.10)	
	Median	3.00	3.00		3.00	3.00	
	Min, Max	0.0, 6.0	0.0, 7.0		0.0, 5.0	0.0, 7.0	
**WOMAC function**	n/n miss	68/0	66/0		64/0	198/0	0.86
	Mean (SEM)	16.09 (1.19)	16.89 (1.02)		16.16 (1.18)	16.38 (0.65)	
	Median	13.00	17.00		15.00	15.00	
	Min, Max	2.0, 43.0	2.0, 39.0		1.0, 41.0	1.0, 43.0	
**VAS pain**	n/n miss	68/0	66/0		64/0	198/0	0.97
	Mean (SEM)	40.28 (2.15)	40.95 (2.29)		40.75 (2.04)	40.66 (1.24)	
	Median	40.00	40.00		40.00	40.00	
	Min, Max	10.0, 85.0	5.0, 80.0		10.0, 80.0	5.0, 85.0	
**SF36**	n/n miss	67/1	66/0		64/0	197/1	0.68
	Mean (SEM)	54.48 (17.90)	53.79 (20.68)		54.30 (19.70)	54.19 (19.35)	
	Median	50.00	50.00		50.00	50.00	
	Min, Max	25.0, 100.0	25.0, 100.0		25.0, 100.0	25.0, 100.0	
**Walking test 20 m (s)**	n/n miss	68/0	66/0		64/0	198/0	0.51
	Mean (SEM)	14.29 (0.25)	14.65 (0.22)		14.58 (0.22)	14.50 (0.14)	
	Median	14.06	14.31		14.54	14.27	
	Min, Max	8.8, 22.2	11.3, 20.2		11.1, 19.9	8.8, 22.2	
**SPPB**	n/n miss	68/0	66/0		64/0	198/0	0.26
	Mean (SEM)	10.50 (0.15)	10.36 (0.15)		10.70 (0.14)	10.52 (0.08)	
	Median	11.00	10.00		11.00	11.00	
	Min, Max	8.0,12.0	6.0, 12.0		9.0, 12.00	6.0, 12.0	
**IPAQ**	n/n miss	64/4		63/3	64/0	193/7	0.71
	Mean (SEM)	2711.58 (400.61)		2996.67 (509.16)	3251.88 (472.38)	2986.66 (266.05)	
	Median	1623.00		1332.00	1721.50	1428.00	
	Min, Max	99.0, 19,640.0		0.0, 23,640.0	198.00,17,695.0	0.0, 23,640.0	

*BMI* Body Mass Index, *WOMAC* Western Ontarion McMaster osteoarthritis index, *VAS* Visual analog scale, *SF-36* Short Form (36), *SPPB* Short Physical Performance Battery, *IPAQ* International Physical Activity Questionnaire. Baseline differences assessed by one-way-analysis-of variance for continuous data, and Pearson’s Chi Square for categorical data

### Clinical outcomes

**Fig. 2:**
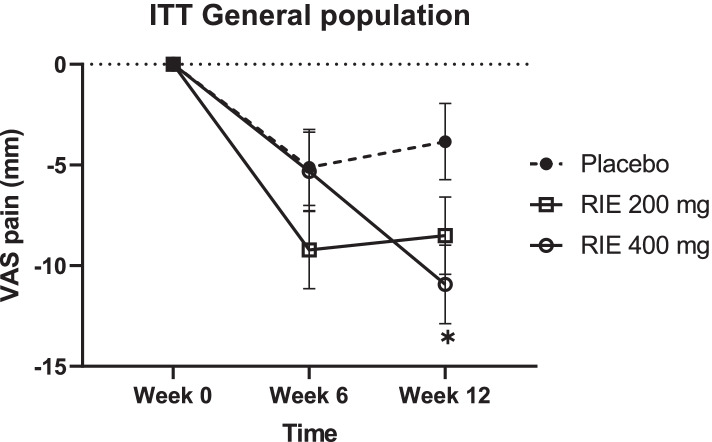
Time evolution of the VAS pain score in ITT population. * = significant effect of RIE 400 mg compared to placebo

In ITT population, WOMAC pain (in mean (SEM) at baseline: 4.89 (0.21)) significantly decreased over time in all groups (*p* < 0.0001). However, there was no difference between treatments. RIE 200 mg and 400 mg after 12 weeks of treatment reduced pain measured by the VAS respectively of -8.51 (1.92) mm and -10.93 (1.95) mm compared to baseline, while the placebo group had a -3.84 (1.89) mm reduction. This means pain reduction induced by RIE reached a statistical difference compared to placebo at the highest dose (-7.09 (2.71); 95% CI, -13.11 to -1.07; *p* = 0,017) (Fig. 2, Table 2). At the daily dosage of 200 mg or 400 mg, the effect size calculated on the VAS pain score of the ITT population was 0.30 and 0.45 after 12 weeks, respectively.

**Table 2:** Primary and secondary outcomes – Absolute change from baseline in ITT population

		6 Weeks			12 Weeks		
		Placebo	RIE 200 mg	RIE 400 mg	Placebo	RIE 200 mg	RIE400 mg
**WOMAC Pain** **(primary outcome)**	Mean (SE) 95% CI P value vs baseline P value vs placebo	1.29 (0.31) -1.89; -0.68 < 0.0001	-1.91 (0.31) -2.52; -1.29 < 0.0001 0.2718	-1.79 (0.32) -2.41; -1.16 < 0.0001 0.4199	-1.98 (0.31) -2.59; -1.36 < 0.0001	-2.01 (0.31) -2.63; -1.40 < 0.0001 0.9955	-2.46 (0.32) -3.09; -1.84 < 0.0001 0.4517
**WOMAC global**	Mean (SE) 95% CI P value vs baseline P value vs placebo	-5.60 (1.52) -8.58; -2.62 0.0002	-7.28 (1.54) -10.31; -4.25 < 0.0001 0.6573	-7.66 (1.57) -10.73; -4.59 < 0.0001 0.5416	-10.22 (1.54) -13.25; -7.20 < 0.0001	-9.41 (1.54) -12.44; -6.38 < 0.0001 0.9036	-11.29 (1.57) -14.36; -8.21 < 0.0001 0.8441
**WOMAC stiffness**	Mean (SE) 95% CI P value vs baseline P value vs placebo	-0.48 (0.17) -0.81; -0.14 0.0052	-0.79 (0.17) -1.13; -0.45 < 0.0001 0.3299	-0.95 (0.17) -1.29; -0.60 < 0.0001 0.0970	-1.14 (0.17 -1.47; -0.80 < 0.0001	-1.08 (0.17) -1.41; -0.74 < 0.0001 0.9587	-1.01 (0.17) -1.35; -0.67 < 0.0001 0.8310
**WOMAC function**	Mean (SE) 95% CI P value vs baseline P value vs placebo	--3.60 (1.08) --5.72; -1.49 0.0009	-4.30 (1.09) -6.45; -2.16 < 0.0001 0.8609	-4.61 (1.11) -6.79; -2.44 < 0.0001 0.7301	-6.69 (1.09) -8.84; -4.55 < 0.0001	-5.95 (1.09) -8.10; -3.81 < 0.0001 0.8480	-7.36 (1.11) -9.54; -5.19 < 0.0001 0.8742
**VAS pain**	Mean (SE) 95% CI P value vs baseline P value vs placebo	-5.12 (1.89) -8.83; -1.42 0.0068	-9.22 (1.92) -12.99; -5.46 < 0.0001 0.2223	-5.32 (1.95) -9.15; -1.50 0.0064 0.9960	-3.84 (1.89) -7.55; -0.14 0.0421	-8.51 (1.92) -12.27; -4.75 < 0.0001 0.1485	-10.93 (1.95) -14.76; -7.11 < 0.0001 **0.0176**
**SF-36**	Mean (SD) Min., Max P value vs placebo	5.97 (16.91) -25.00, 50.00	4.55 (19.07) -50.00, 75.00 0.9761	-1.17 (16.32) -50.00, 50.00 0.0544	2.61 (19.04) -50.00, 50.00	2.27 (21.36) -50.00, 50.00 0.9785	3.13 (20.65) -50.00, 50.00 0.9212
**20 m walking test**	Mean (SD) Min., Max P value vs placebo	-0.31 (1.30) -4.1, 3.2	-0.41 (1.75) -4.0, 7.6 0.9391	-0.36 (1.75) -6.7, 3.5 0.9838	-0.18 (1.53) -4.2, 4.2	-0.39 (1.69) -4.5, 3.0 0.9349	-0.30 (1.94) -6.9, 3.8 0.9724
**SPPB**	Mean (SD) Min., Max P value vs placebo	0.21 (0.86) -2.0, 2.0	0.26 (1.23) -2.0, 6.0 0.9996	0.05 (0.72) -2.0, 2.0 0.5601	0.34 (0.94) -2.0, 2.0	0.39 (1.37) -4.0, 6.0 1.0000	0.22 (0.81) -3.0, 2.0 0.6386
**IPAQ**	Mean (SD) Min., Max P value vs placebo	107.48 (298.81) -90.2, 1693.8	173.08 (560.57) -100.0, 3400.0 0.9493	102.46 (279.33) -89.6, 1300.0 0.9306	38.38 (275.67) -95.0, 2000.0	37.48 (216.29) -94.3, 1339.4 0.9601	36.62 (184.88) -96.5, 1052.0 0.9950

**Fig. 3:**
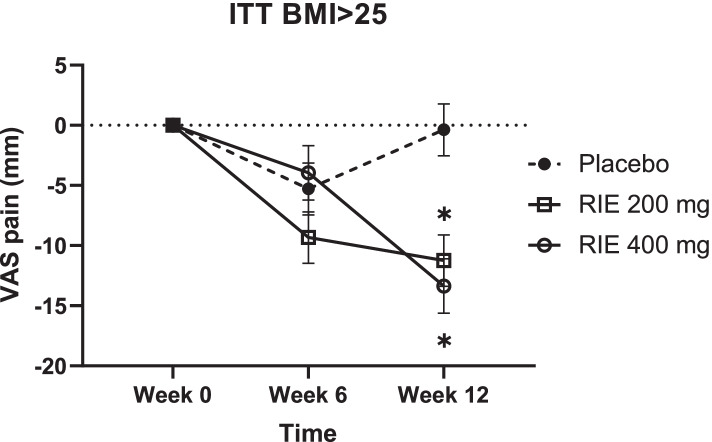
Time evolution of VAS pain in the participant of the ITT population with a Body Mass Index (BMI) > to 25. * = significant effect of RIE compared to placebo

After 12 weeks of treatment, a subgroup analysis of the participants with a BMI ≥ 25, highlighted VAS pain reduction for the 200 and 400 mg doses compared to baseline of respectively -11.25 (2.14) mm and -13.36 (2.26) mm (placebo = -0.37 (2,16) mm). The reduction was statistically different versus placebo for both doses (200 mg -10.88 (3.04); 95% CI, -17.64 to -4.12 p = 0.0008 and 400 mg -12.99 (3.13); 95% CI, -19.95 to -6.04 *p* < 0,0001) (Fig. 3). No significant effect of RIE was observed in the normal BMI group.

The WOMAC global score, stiffness, and physical function subscores decreased significantly with time in all groups. The decrease tended to be more important in the RIE treated groups than in the placebo groups but no significant difference between groups was observed (Table 2). RIE at 400 mg was significantly more efficient than placebo to decrease the WOMAC stiffness score in the normal BMI group after 6, but not 12 weeks of treatment (p = 0.042). In the normal BMI group, a higher decrease in the WOMAC pain, physical function, and global scores were observed in the RIE 200 mg group than in the placebo group after 6 weeks of treatment (WOMAC global pain: *p* = 0.007742; WOMAC physical function: *p* = 0.0027; WOMAC global: *p* = 0.0026) (Additional file 3).

RIE had no significant effect on the other secondary end-points (IPAQ, SF-36, walking distance in treadmill test, SPPB, and evaluation of associated treatments needed to manage OA) except for the IPAQ score at 400 mg in the normal BMI group at 12 weeks (*p* = 0.017).

**Fig. 4:**
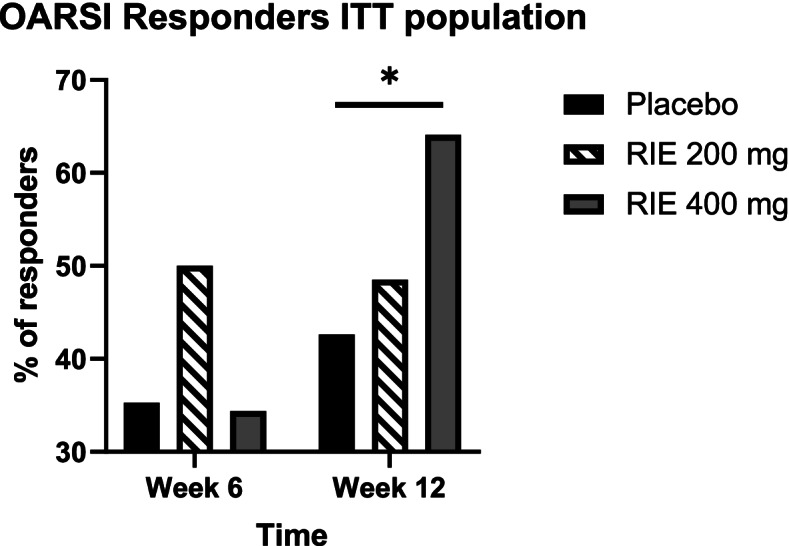
Percentage of participants of the ITT population responding to treatment according to OMERACT-OARSI criteria. * = significant effect of RIE compared to placebo

After 12 weeks of treatment, over 60% of patients fulfilled the OMERACT-OARSI criteria in RIE 400 mg group, but only 45% in the placebo (*p* = 0.04) (Fig. 4).

There were two not-related-to-product Serious Adverse Events (SAE) reported in this study. One participant had a severe SAE as they were diagnosed with prostate cancer. This participant was withdrawn from the trial due to SAE after visit 1. The other participant had a moderate SAE due to renal calculus. This participant recovered within three days and remained in the trial.

There were 123 AEs reported by 87 (42.9%) participants in total for the Safety Population (*N* = 203). In addition, 95.1% of AEs were mild to moderate intensity. There were 29 participants (42.6%) in the placebo group who reported ≥ 1 AE(s), 32 participants (46.4%) in the RIE 200 mg group who reported ≥ 1 AEs, and 26 participants (39.4%) in the RIE 400 mg, who reported ≥ 1 AE(s). However, there were only 22 participants (10.8%) with ≥ 1 AEs possibly-related-to-product in the Safety Population (*N* = 203) who reported a total of 24 AEs possibly-related-to-product. All other AEs reported were identified as not related-to-product. There were four participants (5.9%) in the placebo group who reported 1 AE possibly-related -to-product, ten participants (14.5%) in the RIE 200 mg group who reported ≥ 1 AEs possibly-related-to-product, and eight participants (12.1%) in the RIE 400 mg who reported ≥ 1 AEs possibly-related-to-product. Only one participant in each active product group had reported two possibly-related-to-product AEs. Eight (33.3%) of the possibly-related-to-product AEs (*N* = 24) were due to elevated blood results requiring General Practitioner follow-up reported by eight participants (*N* = 2 Placebo; *N* = 4 RIE 200 mg group; *N* = 2 RIE 400 mg group). Twelve (50.0%) possibly-related-to-product AEs (*N* = 24) were due to gastrointestinal issues reported by ten participants (*N* = 1 Placebo; *N* = 5 RIE 200 mg group; *N* = 4 RIE 400 mg group) where three participants had to discontinue the study due to diarrhea. One of these AEs (RIE 400 mg) was deemed severe for epigastric pain (RIE 400 mg) but this had resolved within one day with no reoccurrence. Two (8.3%) were due to skin rashes (*N* = 2 RIE 400 mg group). The two remaining AEs were a severe AE for a swollen knee (RIE 400 mg) and an AE (Placebo) for Deep Vein Thrombosis which occurred after a flight abroad.

In sum, there were no SAEs related to the product and there was a low proportion of related-to-product AEs in either active product group. In addition, the overall pattern of safety blood panel and vitals results from baseline to end of the intervention indicated no safety concerns. The product can be viewed as tolerable as only 2.2% (*N* = 3) of the active product groups (*N* = 135) had to discontinue the product due to gastrointestinal AE.

In total, 191 participants returned their product by end of the trial and the population had high study product compliance and adherence to protocol. Of the participants who returned the product, 185 (96.9%) had a study product consumption compliance equal to or greater than 80%. The mean compliance was 95.96% (SD 7.12, Min 57.0%, max 110.0%).

Rescue medication use was recorded in the daily e-diary app as an exploratory outcome. This analysis focused on the week before baseline and week 12 (Table 3). Due to missing data, 21 participants from the ITT population were not included in the analysis for rescue medication use. For those with data, 61.0% (Total *N* = 108; Placebo *N* = 33; Active product *N* = 75) took no rescue medication before baseline to the end of the study, and 22.0% (Total *N* = 39; Placebo *N* = 15; Active product *N* = 24) reduced their use of rescue medication, 13.6% (Total *N* = 24; Placebo *N* = 11; Active product *N* = 13) increased their use of rescue medication and 3.4% (Total *N* = 6; Placebo *N* = 3; Active product *N* = 3) used the rescue medication at the same frequency that at baseline and week 12. The active product group (either dose) attended to have a higher ratio (61.5%; *N* = 24) of reducing their rescue medication use compared to the placebo (38.5%; *N* = 15).

**Table 3:** Rescue Medication Use the week prior to baseline and end of intervention in the ITT population for participants with available data (*N* = 177)

		N	%
Placebo (*N* = 62)	No medication use at either Week 0 or Week 12	33	53.2%
	Medication use reduces from Week 0 to Week 12	15	24.2%
	Medication use increases from Week 0 to Week 12	11	17.7%
	No change between Week 0 and Week 12	3	4.8%
Treatment (200 dose) (*N* = 59)	No medication used at either Week 0 or Week 12	43	72.9%
	Medication use reduces from Week 0 to Week 12	11	18.6%
	Medication use increases from Week 0 to Week 12	2	3.4%
	No change between Week 0 and Week 12	3	5.1%
Treatment (400 dose) (*N* = 56)	No medication used at either Week 0 or Week 12	32	57.1%
	Medication use reduces from Week 0 to Week 12	13	23.2%
	Medication use increases from Week 0 to Week 12	11	19.6%
	No change between Week 0 and Week 12	0	0.0%
Treatment	No medication used at either Week 0 or Week 12	75	65.2%
(*N* = 115)	Medication use reduces from Week 0 to Week 12	24	20.9%
	Medication use increases from Week 0 to Week 12	13	11.3%
	No change between Week 0 and Week 12	3	2.6%

## Discussion

In this paper, we report the data of a clinical trial investigating the clinical efficacy of two doses of RIE, administered orally for 3 months in patients with symptomatic established knee OA. Compared to placebo, RIE was not effective on the WOMAC pain which was the primary end-point. In contrast, RIE significantly and rapidly relieved pain evaluated by VAS in knee OA patients. At the daily dosage of 200 mg or 400 mg, the effect size calculated on the VAS pain score of the ITT population was 0.30 and 0.45 after 12 weeks, respectively. Compared to NSAIDs and paracetamol, the effect size for the pain of RIE is comparable. Indeed, a meta-analysis has reported effect sizes for pain compared to oral placebo comprised between 0.38 and 0.52 for NSAIDs after 12 weeks of treatment [10]. Comparing to paracetamol (ES: 0.18 (0.04 to 0.33), RIE was even more efficient [10]. Considering the excellent safety of RIE, this extract could be a good alternative to NSAIDs and paracetamol that show severe adverse effects after long-term administration. Further, our study demonstrated that RIE treatment was associated with a greater reduction of rescue medications including paracetamol and NSAIDS than placebo. This data again indicates that RIE has an antalgic effect superior to that of paracetamol and NSAIDs. A feature of this study was that it included participants with mild to moderate pain not adequately or completely controlled with NSAIDs. This finding indicates that RIE is efficient where NSAIDS are not. This could be explained by the difference in the mechanisms of action. NSAIDs act on inflammation mainly by inhibiting cyclooxygenases while RIE acts by preventing the activation of MAPK or NFkB signaling pathways that lead to the secretion of a large panel of pro-inflammatory cytokines and metalloproteases [3] as well as on the resolution of the inflammation. We can speculate that in some participants RIE mechanisms of action are more appropriate to relieve symptoms in chronic inflammatory conditions than NSAIDs. This was already observed with other polyphenols like curcumin [11]. Interestingly, a subgroup analysis showed that RIE effect on VAS pain was significant only in overweight/obese participants. Our study fails to bring an explanation to that finding. Both populations were similar in terms of pain or physical activity level at baseline. The only difference was the sex ratio. There were proportionally more men in the overweight/obese group. One hypothesis would be that men are better responders than women to RIE treatment. This needs to be confirmed as we have not observed a difference in RIE efficacy between men and women in the overall population. Another possible explanation would be that RIE through its anti-inflammatory properties acts on systemic inflammation which is associated with obesity. Inflammatory biomarkers should be explored to verify this hypothesis.

Globally, this study also showed that 400 mg per day of RIE is the adequate posology to relieve VAS pain. It is at this dose that we recorded the most responders according to the OMERACT-OARSI criterion. We can also conclude that three months of treatment are necessary to obtain significant analgesia.

This study showed promising effects of RIE on symptoms of knee OA but should also be interpreted with caution because our study suffers from some limitations. First, RIE was not efficient on the primary outcome. The second main limitation was the small sample size as larger groups will be required to confirm the positive findings observed in this study [12].

## Conclusions

This randomized controlled clinical trial demonstrated that RIE, a rubus ideaus extract, is a safe and efficient treatment to manage symptoms of patients with knee OA. Of course, this result needs to be confirmed in a larger phase III clinical trial including not only clinical parameters but also biochemical markers of inflammation and cartilage degradation and imaging structural analysis of joint tissues. This trial provides useful information for the design of a larger phase III clinical trial including the sample size estimate, the choice of the dose, and the selection of primary outcomes.

## Supplementary Information

**Additional file 1. **Listing 1. By Participant Listing Of Analysis Sets.

**Additional file 2. **Comparison of demographic and clinical outcomes atbaseline between normal BMI and overweight/obese BMI group in the ITTpopulation (*N*=198).

**Additional file 3. **Absolute change from baselinein normal BMI group.

**Additional file 4.** Absolute change from baseline in BMI ≥ 25group.

## Data Availability

All data are available and can be obtained by sending a request by mail to Naturex SA, Avignon, France, or by e-mail to romain.le_cozannet@givaudan.com.

## Abbreviations

AE: Adverse effect
BMI: Body Mass Index
COX: Cyclooxygenase
ES: Effect Size
HETE: Hydroxyeicosatetraenoic acids
IPAQ: International Physical Activity Questionnaire
ITT: Intention-To-Treat
MAPK: Mitogen-activated protein kinases
MET: Metabolic Equivalent of Task
MMP: Matrix MetalloProteinase
NFKB: Nuclear Factor-Kappa B
NSAIDs: Nonsteroidal Anti-inflammatory Drugs
OA: Osteoarthritis
OARSI: Osteoarthritis Research Society International
OMERACT: Outcome Measures in Rheumatology
RIE: Rubaeus Ideaus Extract
SEM: Standard Error of Mean
SF-36: Short Form (36)
SPPB: Short Physical Performance Battery
VAS: Visual Analog Scale
WOMAC: Western Ontario McMaster osteoarthritis index
