# Supplementary material for: Rubus idaeus extract improves symptoms in knee osteoarthritis patients: results from a phase II double-blind randomized controlled trial
Source: BMC Musculoskelet Disord. 2022 Jul 7;23:650. doi: 10.1186/s12891-022-05612-2 (PMC9261022; doi:10.1186/s12891-022-05612-2)
Supplement: Supplementary file 1 — Additional file 1. Listing 1. By Participant Listing Of Analysis Sets. [file 12891_2022_5612_MOESM1_ESM.docx]

| Listing 1. By Participant Listing Of Analysis Sets | | | | |
| --- | --- | --- | --- | --- |
| Participant ID | Product Allocation | Safety Population | ITT population | PP population |
|  |  |  |  |  |
| 070-001 | Placebo | I | I | I |
| 070-002 | 400mg Active | I | I | I |
| 070-003 | 200mg Active | I | I |  |
| 070-005 | 400mg Active | I | I | I |
| 070-006 | 200mg Active | I | I |  |
| 070-007 | Placebo | I | I | I |
| 070-008 | 200mg Active | I | I | I |
| 070-009 | Placebo | I | I | I |
| 070-010 | 400mg Active | I | I |  |
| 070-011 | Placebo | I | I | I |
| 070-012 | 200mg Active | I | I | I |
| 070-013 | 400mg Active | I | I |  |
| 070-014 | 200mg Active | I | I |  |
| 070-015 | 400mg Active | I | I | I |
| 070-016 | Placebo | I | I |  |
| 070-017 | Placebo | I | I | I |
| 070-018 | 400mg Active | I | I | I |
| 070-019 | 200mg Active | I | I | I |
| 070-020 | 200mg Active | I | I | I |
| 070-021 | Placebo | I | I |  |
| 070-022 | 400mg Active | I | I |  |
| 070-023 | Placebo | I | I | I |
| 070-024 | 200mg Active | I | I |  |
| 070-025 | 400mg Active | I | I | I |
| 070-028 | 200mg Active | I | I |  |
| 070-029 | 400mg Active | I | I | I |
| 070-030 | Placebo | I | I | I |
| 070-031 | 400mg Active | I | I |  |
| 070-032 | Placebo | I | I |  |
| 070-033 | 200mg Active | I | I | I |
| 070-034 | 200mg Active | I | I |  |
| 070-035 | Placebo | I | I | I |
| 070-036 | 400mg Active | I | I | I |
| 070-038 | 400mg Active | I | I | I |
| 070-039 | 200mg Active | I |  |  |
| 070-040 | Placebo | I | I | I |
| 070-041 | 200mg Active | I | I | I |
| 070-043 | Placebo | I | I | I |
| 070-047 | 400mg Active | I | I |  |
| 070-048 | 200mg Active | I | I | I |
| 070-049 | 400mg Active | I | I | I |
| 070-050 | Placebo | I | I |  |
| 070-051 | Placebo | I | I |  |
| 070-054 | 200mg Active | I | I | I |
| 070-055 | 400mg Active | I | I | I |
| 070-056 | 200mg Active | I | I | I |
| 070-057 | Placebo | I | I | I |
| 070-058 | 400mg Active | I | I | I |
| 070-059 | Placebo | I | I |  |
| 070-060 | 200mg Active | I | I | I |
| 070-061 | 400mg Active | I | I | I |
| 070-062 | 400mg Active | I | I | I |
| 070-063 | Placebo | I | I | I |
| 070-064 | 200mg Active | I | I | I |
| 070-065 | 200mg Active | I | I | I |
| 070-066 | Placebo | I | I | I |
| 070-069 | 400mg Active | I | I | I |
| 070-070 | Placebo | I | I |  |
| 070-071 | 400mg Active | I | I | I |
| 070-072 | 200mg Active | I | I | I |
| 070-073 | 200mg Active | I | I | I |
| 070-074 | Placebo | I | I |  |
| 070-075 | 400mg Active | I | I | I |
| 070-076 | Placebo | I | I | I |
| 070-077 | 200mg Active | I | I | I |
| 070-079 | 400mg Active | I | I | I |
| 070-080 | 400mg Active | I | I | I |
| 070-081 | Placebo | I | I | I |
| 070-082 | 200mg Active | I | I |  |
| 070-083 | 200mg Active | I | I | I |
| 070-085 | 400mg Active | I | I | I |
| 070-086 | Placebo | I | I | I |
| 070-087 | Placebo | I | I | I |
| 070-089 | 200mg Active | I | I | I |
| 070-090 | 400mg Active | I | I | I |
| 070-091 | 400mg Active | I | I | I |
| 070-092 | Placebo | I | I | I |
| 070-093 | Placebo | I | I | I |
| 070-094 | 400mg Active | I | I |  |
| 070-095 | 200mg Active | I | I | I |
| 070-096 | Placebo | I | I | I |
| 070-097 | 200mg Active | I | I | I |
| 070-098 | 200mg Active | I | I | I |
| 070-100 | Placebo | I | I | I |
| 070-101 | 200mg Active | I | I | I |
| 070-102 | 400mg Active | I | I | I |
| 070-103 | Placebo | I | I | I |
| 070-105 | 200mg Active | I | I | I |
| 070-106 | 400mg Active | I | I | I |
| 070-108 | 400mg Active | I | I | I |
| 070-109 | 200mg Active | I | I | I |
| 070-110 | 400mg Active | I | I | I |
| 070-111 | Placebo | I | I | I |
| 070-112 | 200mg Active | I | I | I |
| 070-113 | Placebo | I | I | I |
| 070-114 | 400mg Active | I | I | I |
| 070-115 | Placebo | I | I | I |
| 070-116 | 200mg Active | I | I | I |
| 070-117 | Placebo | I | I |  |
| 070-118 | 400mg Active | I | I | I |
| 070-119 | 200mg Active | I | I | I |
| 070-121 | 400mg Active | I | I | I |
| 070-123 | 200mg Active | I | I |  |
| 070-124 | Placebo | I | I | I |
| 070-125 | Placebo | I | I | I |
| 070-126 | 200mg Active | I | I | I |
| 070-127 | 400mg Active | I | I | I |
| 070-128 | Placebo | I | I | I |
| 070-130 | 200mg Active | I | I | I |
| 070-131 | 400mg Active | I | I | I |
| 070-132 | 200mg Active | I | I | I |
| 070-133 | Placebo | I | I | I |
| 070-134 | 400mg Active | I | I | I |
| 070-135 | Placebo | I | I | I |
| 070-136 | 200mg Active | I | I | I |
| 070-137 | 400mg Active | I | I | I |
| 070-139 | Placebo | I | I | I |
| 070-140 | 400mg Active | I | I | I |
| 070-141 | 200mg Active | I | I | I |
| 070-142 | 400mg Active | I | I | I |
| 070-143 | 200mg Active | I | I | I |
| 070-144 | Placebo | I | I | I |
| 070-145 | Placebo | I | I | I |
| 070-146 | 200mg Active | I | I | I |
| 070-147 | 400mg Active | I |  |  |
| 070-148 | Placebo | I | I | I |
| 070-149 | 400mg Active | I | I | I |
| 070-150 | 200mg Active | I | I | I |
| 070-151 | Placebo | I | I | I |
| 070-152 | 200mg Active | I |  |  |
| 070-153 | 400mg Active | I | I | I |
| 070-154 | 400mg Active | I | I | I |
| 070-156 | Placebo | I | I | I |
| 070-157 | 200mg Active | I | I | I |
| 070-158 | Placebo | I | I | I |
| 070-159 | 200mg Active | I | I | I |
| 070-160 | 400mg Active | I | I |  |
| 070-161 | Placebo | I | I | I |
| 070-162 | 400mg Active | I | I | I |
| 070-163 | 200mg Active | I | I | I |
| 070-164 | 400mg Active | I | I | I |
| 070-165 | 200mg Active | I | I |  |
| 070-166 | Placebo | I | I | I |
| 070-167 | 200mg Active | I | I | I |
| 070-169 | Placebo | I | I | I |
| 070-170 | 400mg Active | I | I | I |
| 070-171 | Placebo | I | I | I |
| 070-172 | 200mg Active | I | I | I |
| 070-173 | 400mg Active | I | I |  |
| 070-174 | Placebo | I | I | I |
| 070-175 | 400mg Active | I | I | I |
| 070-176 | 200mg Active | I | I | I |
| 070-177 | Placebo | I | I |  |
| 070-178 | 400mg Active | I | I | I |
| 070-179 | 200mg Active | I |  |  |
| 070-180 | Placebo | I | I |  |
| 070-181 | 400mg Active | I | I | I |
| 070-182 | 200mg Active | I | I | I |
| 070-184 | 200mg Active | I | I |  |
| 070-185 | Placebo | I | I |  |
| 070-186 | 200mg Active | I | I |  |
| 070-187 | Placebo | I | I | I |
| 070-188 | 400mg Active | I | I |  |
| 070-189 | 400mg Active | I | I | I |
| 070-190 | 200mg Active | I | I | I |
| 070-191 | Placebo | I | I |  |
| 070-192 | 200mg Active | I | I | I |
| 070-193 | 400mg Active | I | I | I |
| 070-195 | 400mg Active | I | I |  |
| 070-196 | Placebo | I | I | I |
| 070-197 | 200mg Active | I | I | I |
| 070-198 | 400mg Active | I | I |  |
| 070-199 | Placebo | I | I | I |
| 070-200 | 200mg Active | I | I | I |
| 070-201 | Placebo | I | I | I |
| 070-202 | 400mg Active | I |  |  |
| 070-203 | 200mg Active | I | I |  |
| 070-204 | 400mg Active | I | I | I |
| 070-205 | 200mg Active | I | I | I |
| 070-206 | Placebo | I | I | I |
| 070-207 | 200mg Active | I | I | I |
| 070-210 | 400mg Active | I | I | I |
| 070-211 | Placebo | I | I | I |
| 070-212 | Placebo | I | I |  |
| 070-213 | 200mg Active | I | I | I |
| 070-214 | 400mg Active | I | I | I |
| 070-215 | Placebo | I | I | I |
| 070-216 | 200mg Active | I | I | I |
| 070-217 | 400mg Active | I | I |  |
| 070-218 | Placebo | I | I | I |
| 070-219 | 400mg Active | I | I |  |
| 070-220 | 200mg Active | I | I |  |
| 070-221 | 200mg Active | I | I | I |
| 070-222 | 400mg Active | I | I | I |
| 070-223 | Placebo | I | I |  |
| 070-224 | 400mg Active | I | I | I |
| 070-225 | Placebo | I | I | I |
| 070-226 | 200mg Active | I | I | I |
| 070-228 | Placebo | I | I |  |
| 070-229 | 200mg Active | I | I |  |
| 070-231 | Placebo | I | I | I |
| 070-232 | 400mg Active | I | I | I |
| 070-233 | 200mg Active | I | I | I |
