# Supplementary material for: Rubus idaeus extract improves symptoms in knee osteoarthritis patients: results from a phase II double-blind randomized controlled trial
Source: BMC Musculoskelet Disord. 2022 Jul 7;23:650. doi: 10.1186/s12891-022-05612-2 (PMC9261022; doi:10.1186/s12891-022-05612-2)
Supplement: Supplementary file 2 — Additional file 2. Comparison of demographic and clinical outcomes atbaseline between normal BMI and overweight/obese BMI group in the ITTpopulation (N=198). [file 12891_2022_5612_MOESM2_ESM.docx]

| **Additional file 2. Comparison of demographic and clinical outcomes at baseline between normal BMI and overweight/obese BMI group in the ITT population (N=198).** | | | | | | | | | |
| --- | --- | --- | --- | --- | --- | --- | --- | --- | --- |
|  | |  | | **BMI**  **<25 kg/m^2^** | | **BMI**  **≥ 25 kg/m^2^** | | **All**  **(N=198)** | **P-values** |
| **Sex** | | n/n miss | | 51/0 | | 147/0 | | | 198/0 |
|  | Male, n(%) | | | 12 (23.5%) | | 62 (42.2%) | | 74 (37.4%) | 0.02 |
|  | Female, n(%) | | | 39 (76.5%) | | 85 (57.8%) | | 124 (62.6%) |  |
| **Age (years)** | n/n miss | | | 51/0 | | 147/0 | | 198/0 | 0.89 |
|  | Mean (SEM) | | | 54.92 (1.68) | | 54.69 (0.82) | | 54.75 (0.75) |  |
|  | Median | | | 54.00 | | 55.00 | | 55.00 |  |
|  | Min, Max | | | 30.0, 75.0 | | 32.0, 74.0 | | 30.0, 75.0 |  |
| **Total Body Weight (kg)** | n/n miss | | | 51/0 | | 147/0 | | 198/0 | <0.0005 |
|  | Mean (SEM) | | | 65.05 (1.24) | | 81.96 (0.92) | | 77.60 (0.92) |  |
|  | Median | | | 63.40 | | 81.40 | | 77.20 |  |
|  | Min, Max | | | 49.2, 88.,4 | | 58.7, 116.7 | | 49.2, 116.7 |  |
| **BMI (kg/m^2^)** | n/n miss | | | 51/0 | | 147/0 | | 198/0 | <0.0005 |
|  | Mean (SEM) | | | 22.92 (0.19) | | 28.30 (0.19) | | 26.92 (0.22) |  |
|  | Median | | | 23.33 | | 28.13 | | 26.69 |  |
|  | Min, Max | | | 19.06, 24.41 | | 24.59, 33.88 | | 19.06, 33.88 |  |
| **WOMAC global** | n/n miss | | | 51/0 | | 147/0 | | 198/0 | 0.52 |
|  | Mean (SEM) | | | 24.02 (1.76) | | 25.37 (1.07) | | 25.02 (0.91) |  |
|  | Median | | | 21.88 | | 22.92 | | 22.92 |  |
|  | Min, Max | | | 7.29, 50.00 | | 3.13, 63.54 | | 3.1, 63.5 |  |
| **WOMAC pain** | n/n miss | | | 51/0 | | 147/0 | | 198/0 | 0.36 |
|  | Mean (SEM) | | | 5.22 (0.37) | | 4.78 (0.25) | | 4.89 (0.21) |  |
|  | Median | | | 5.00 | | 4.00 | | 4.00 |  |
|  | Min, Max | | | 1.0, 12.0 | | 0.0, 16.0 | | 0.0, 16.0 |  |
| **WOMAC stiffness** | n/n miss | | | 51/0 | | 147/0 | | 198/0 | 0.07 |
|  | Mean (SEM) | | | 2.45 (0.18) | | 2.86 (0.12) | | 2.75 (0.10) |  |
|  | Median | | | 2.00 | | 3.00 | | 3.00 |  |
|  | Min, Max | | | 0.0, 5.0 | | 0.0, 7.0 | | 0.0, 7.0 |  |
| **WOMAC function** | n/n miss | | | 51/0 | | 147/0 | | 198/0 | 0.37 |
|  | Mean (SEM) | | | 15.39 (1.30) | | 16.72 (0.75) | | 16.38 (0.65) |  |
|  | Median | | | 12.00 | | 15.00 | | 15.00 |  |
|  | Min, Max | | | 4.0, 35.0 | | 1.0, 43.0 | | 1.0, 43.0 |  |
| **VAS pain** | n/n miss | | | 51/0 | | 147/0 | | 198/0 | 0.30 |
|  | Mean (SEM) | | | 38.45 (2.33) | | 41.42 (1.46) | | 40.66 (1.24) |  |
|  | Median | | | 2.33 | | 40.00 | | 40.00 |  |
|  | Min, Max | | | 10.0, 83.0 | | 5.0, 85.0 | | 5.0, 85.0 |  |
| **SPPB** | n/n miss | | | 51/0 | | 147/0 | | 198/0 | 0.95 |
|  | Mean (SEM) | | | 10.53 (0.16) | | 10.52 (0.10) | | 10.52 (0.08) |  |
|  | Median | | | 11.00 | | 11.00 | | 11.00 |  |
|  | Min, Max | | | 8.0, 12.0 | | 6.0, 12.0 | | 6.0, 12.0 |  |
| **IPAQ** | n/n miss | | | 49/2 | | | 142/5 | 191/7 | 0.53 |
|  | Mean (SEM) | | | 3271.34 (452.12) | | | 2888.42 (322.52) | 2986.66 (266.05) |  |
|  | Median | | | 2700.00 | | | 1386.00 | 1428.00 |  |
|  | Min, Max | | | 0.0, 16768.0 | | |  | 0.0, 23640.0 |  |
