# Supplementary material for: Rubus idaeus extract improves symptoms in knee osteoarthritis patients: results from a phase II double-blind randomized controlled trial
Source: BMC Musculoskelet Disord. 2022 Jul 7;23:650. doi: 10.1186/s12891-022-05612-2 (PMC9261022; doi:10.1186/s12891-022-05612-2)
Supplement: Supplementary file 3 — Additional file 3. Absolute change from baselinein normal BMI group. [file 12891_2022_5612_MOESM3_ESM.docx]

| **Additional file 3. Absolute change from baseline in normal BMI group** | | | | | | | |
| --- | --- | --- | --- | --- | --- | --- | --- |
| **Normal BMI group** |  | 6 Weeks | | | 12 Weeks | | |
|  |  | Placebo | RIE 200 mg | RIE 400 mg | Placebo | RIE 200 mg | RIE400 mg |
| **WOMAC Pain** | Mean (SE)  95% CI  P value vs baseline  P value vs placebo | -0.61 (0.52)  -1.63 ; 0.41  0.2364 | -2.88 (0.57)  -4.00 ; -1.76  <0.0001  **0.0077** | -1.29 (0.52)  -2.31 ; -0.27  0.0138  0.5571 | -2.08 (0.54)  -3.15 ; -1.00  0.0002 | -2.70 (0.57)  -3.82 ; -1.58  <0.0001  0.6424 | -2.24 (0.52)  -3.26 ; -1.22  <0.0001  0.9661 |
| **WOMAC global** | Mean (SE)  95% CI  P value vs baseline  P value vs placebo | 1.19 (2.28)  -3.32 ; 5.70  0.6028 | -10.05 (2.50)  -14.99 ; -5.11  <0.0001  **0.0026** | -4.56 (2.28)  -9.05 ; -0.06  0.0471  0.1372 | -5.65 (2.41)  -10.40 ; -0.89  0.0202 | -10.54 (2.50)  -15.48 ; -5.60  <0.0001  0.2792 | -9.92 (2.28)  -14.41 ; -5.42  <0.0001  0.3304 |
| **WOMAC stiffness** | Mean (SE)  95% CI  P value vs baseline  P value vs placebo | 0.41 (0.30)  -0.18 ; 1.00  0.1711 | -0.11 (0.32)  -0.74 ; 0.53  0.7393  0.3970 | -0.57 (0.30)  -1.15 ; 0.02  0.0586  **0.0421** | -0.65 (0.31)  -1.27 ; -0.03  0.0393 | -0.81 (0.32)  -1.45 ; -0.18  0.0126  0.9111 | -0.97 (0.30)  -1.55 ; -0.38  0.0014  0.6890 |
| **WOMAC function** | Mean (SE)  95% CI  P value vs baseline  P value vs placebo | 1.25 (1.59)  -1.89 ; 4.39  0.4333 | -6.55 (1.74)  -9.99 ; -3.11  0.0002  **0.0027** | -2.52 (1.58)  -5.65 ; 0.61  0.1140  0.1686 | -2.81 (1.68)  -6.12 ; 0.50  0.0961 | -6.49 (1.74)  -9.93 ; -3.05  0.0003  0.2300 | -6.32 (1.58)  -9.45 ; -3.19  0.0001  0.2233 |
| **VAS pain** | Median  Q1 ; Q3  P value vs placebo | -7.50  -14.50 ; 5.50 | -5.00  -15.00 ; 0.00  0.9229 | -10.00  -17.50 ; 0.00  0.9855 | -12.00  -27.50 ; -1.00 | 2.00  -20.00 ; 13.00  0.2389 | -5.50  -20.50;10.50  0.4420 |
| **SF-36** | Mean (SD)  Ratio from baseline  P value vs placebo | 6.25 (15.97)  1.15 | 7.35 (22.99)  1.19  0.8760 | -1.25 (12.76)  1.03  0.3036 | 8.75 (18.63)  1.17 | -2.94 (19.53)  0.98  0.0648 | 6.25 (24.16)  1.17  0.9997 |
| **20 m walking test** | Median  Q1 ; Q3  P value vs placebo | -0.36  -0.80 ; 0.86 | -0.32  -0.81 ; 0.35  0.9873 | -0.19  -0.82 ; 0.29  0.9642 | 0.06  -0.89 ; 0.98 | -0.17  -0.92 ; 0.58  0.8313 | -0.14  -0.88 ; 0.83  0.9605 |
| **SPPB** | Mean (SE)  95% CI  P value vs placebo | 0.16 (0.15)  -0.13 ; 0.45 | -0.02 (0.16)  -0.33 ; 0.30  0.6299 | 0.21 (0.15)  -0.08 ; 0.49  0.9654 | 0.46 (0.15)  0.17 ; 0.75 | 0.10 (0.16)  -0.21 ; 0.41  0.1750 | 0.36 (0.15)  0.07 ; 0.64  0.8337 |
| **IPAQ** | Median  Q1 ; Q3  P value vs placebo | -358.00  -1316 ; 336 | -181.50  -896.25;1150.5  0.9823 | -1879.25  -3432.75 ; 229  0.1417 | -150.00  -880 ; 414 | -619.50  -1441.5 ; 240  0.8567 | -2783.25  -3678 ; -906.5  **0.0176** |
