# Supplementary material for: Rubus idaeus extract improves symptoms in knee osteoarthritis patients: results from a phase II double-blind randomized controlled trial
Source: BMC Musculoskelet Disord. 2022 Jul 7;23:650. doi: 10.1186/s12891-022-05612-2 (PMC9261022; doi:10.1186/s12891-022-05612-2)
Supplement: Supplementary file 4 — Additional file 4. Absolute change from baseline in BMI ≥ 25group. [file 12891_2022_5612_MOESM4_ESM.docx]

| **Additional file 4. Absolute change from baseline in BMI ≥ 25 group** | | | | | | | |
| --- | --- | --- | --- | --- | --- | --- | --- |
| **BMI ≥ 25 group** |  | 6 Weeks | | | 12 Weeks | | |
|  |  | Placebo | RIE 200 mg | RIE 400 mg | Placebo | RIE 200 mg | RIE400 mg |
| **WOMAC Pain** | Mean (SE)  95% CI  P value vs baseline  P value vs placebo | -1.55 (0.38)  -2.30 ; -0.80  <0.0001 | -1.62 (0.38)  -2.36 ; -0.88  <0.0001  0.9875 | -1.98 (0.40)  -2.76 ; -1.20  <0.0001  0.6507 | -1.93 (0.38)  -2.67 ; -1.18  <0.0001 | -1.82 (0.38)  -2.56 ; -1.09  <0.0001  0.9743 | -2.53 (0.40)  -3.31 ; -1.75  <0.0001  0.4444 |
| **WOMAC global** | Mean (SE)  95% CI  P value vs baseline  P value vs placebo | -8.05 (1.89)  -11.77 ; -4.33  <0.0001 | -6.98 (1.87)  -10.66 ; -3.29  0.0002  0.8898 | -8.76 (1.98)  -12.64 ; -4.87  <0.0001  0.9530 | -11.57 (1.89)  -15.29 ; -7.84  <0.0001 | -9.68 (1.87)  -13.36 ; -5.99  <0.0001  0.7021 | -11.60 (1.98)  -15.48 ; -7.71  <0.0001  0.9999 |
| **WOMAC stiffness** | Mean (SE)  95% CI  P value vs baseline  P value vs placebo | -0.84 (0.20)  -1.24 ; -0.43  <0.0001 | -1.04 (0.20)  -1.44 ; -0.64  <0.0001  0.7113 | -1.11 (0.21)  -1.54 ; -0.69  <0.0001  0.5460 | -1.32 (0.20)  -1.72 ; -0.91  <0.0001 | -1.18 (0.20)  -1.58 ; -0.78  <0.0001  0.8521 | -1.02 (0.21)  -1.44 ; -0.60  <0.0001  0.5127 |
| **WOMAC function** | Mean (SE)  95% CI  P value vs baseline  P value vs placebo | -5.32 (1.34)  -7.95 ; -2.68  <0.0001 | -4.04 (1.33)  -6.65 ; -1.43  0.0025  0.7248 | -5.33 (1.40)  -8.09 ; -2.58  0.0002  0.9999 | -7.84 (1.34)  -10.47 ; -5.20  <0.0001 | -6.29 (1.33)  -8.90 ; -3.68  <0.0001  0.6250 | -7.61 (1.40)  -10.36 ; -4.85  <0.0001  0.9897 |
| **VAS pain** | Median  Q1 ; Q3  P value vs placebo | -5.29 (2.16)  -9.54 ; -1.04  0.0148 | -9.33 (2.14)  -13.53 ; -5.13  <0.0001  0.3106 | -3.95 (2.26)  -8.39 ; 0.48  0.0804  0.8770 | -0.37 (2.16)  -4.62 ; 3.88  0.8636 | -11.25 (2.14)  -15.45 ; -7.05  <0.0001  **0.0008** | -13.36 (2.26)  -17.80 ; -8.93  <0.0001  **<0.0001** |
| **SF-36** | Mean (SD)  Ratio from baseline  P value vs placebo | 5.85 (17.46)  1.14  0.0006 | 3.57 (17.68)  1.08  0.0283  0.5537 | -1.14 (17.85)  0.97  0.4620  **0.0072** | 0.00 (18.80)  1.02  0.5793 | 4.08 (21.86)  1.09  0.0130  0.3011 | 1.70 (18.98)  1.02  0.6911  0.9927 |
| **20 m walking test** | Mean (SE)  95% CI  P value vs placebo | -0.46 (0.17)  -0.80 ; -0.12 | -0.60 (0.17)  -0.94 ; -0.27  0.7701 | -0.19 (0.18)  -0.55 ; 0.16  0.4678 | -0.39 (0.17)  -0.73 ; -0.05 | -0.44 (0.17)  -0.78 ; -0.11  0.9657 | -0.23 (0.18)  -0.58 ; 0.13  0.7345 |
| **SPPB** | Mean (SE)  95% CI  P value vs placebo | 0.00  0.00 ; 1.00 | 0.00  0.00 ; 1.00  0.9978 | 0.00  0.00 ; 0.00  0.4106 | 0.00  0.00 ; 1.00 | 0.00  0.00 ; 1.00  0.8532 | 0.00  0.00 ; 1.00  0.8104 |
| **IPAQ** | Median  Q1 ; Q3  P value vs placebo | 324.00  -1515 ; 1470 | 0.00  -971 ; 1296  0.9327 | 91.50  -784.5 ; 1863  0.9733 | -588.00  -2244 ; 282.5 | -285.75  -2277 ; 365  0.9246 | 1.00  -927 ; 876  0.1774 |
